# The Effect of Whole-Grain Diet on the Gut Microbiota of the Elderly Individuals

**DOI:** 10.3389/fnut.2022.919838

**Published:** 2022-06-27

**Authors:** Zeying Cui, Jingtai Li, Yuting Zhen, Pingming Fan, Guankui Du

**Affiliations:** ^1^Department of Breast Surgery, The First Affiliated Hospital of Hainan Medical University, Haikou, China; ^2^Key Laboratory of Molecular Biology, Hainan Medical University, Haikou, China; ^3^Department of Biochemistry and Molecular Biology, Hainan Medical University, Haikou, China; ^4^Biotechnology and Biochemistry Laboratory, Hainan Medical University, Haikou, China

**Keywords:** gut microbiota, whole grain, American Gut Program, overweight, female

## Abstract

A whole-grain (WG) diet affects human health in multiple ways. However, the effect of WG on the gut microbiota of the elderly individuals is still largely unknown. In this study, WG did not affect the microbial α-diversity but had a profound impact on the microbes' abundance in the elderly individuals. WG increased the abundance of *Verrucomicrobia* and decreased the abundance of *Firmicutes*. The prediction of microbial function showed that glucose metabolism and lipid metabolism were inhibited. In addition, the effects of WG on the gut microbiota of normal-weight (NW) and overweight (OW) individuals were different. WG increased *Verrucomicrobia* in the NW group and decreased *Firmicutes* in the OW group. Meanwhile, the effect of WG on gut microbiota showed gender characteristics, *Firmicutes/Bacteroidetes* ratio was decreased in women, while *Verrucomicrobia* abundance was increased in men. The use of WG could improve the microbial composition and promote the growth of beneficial microbes, which may be beneficial to the health of the elderly individuals.

## Introduction

Healthy aging is the key to alleviating the pressure of population aging (1). With the increase of age, the physiological functions of the various organs of the elderly individuals would significantly decrease. The metabolic function of the elderly individuals would be disordered, and their immunity would be significantly decreased (2). The elderly individuals are prone to various kinds of chronic diseases, such as hypertension, diabetes, coronary heart disease, and cancer (3, 4). A healthy lifestyle is important for the health of the elderly individuals (5). The most direct way of health management is to intervene in the diet of the elderly individuals.

Studies have shown that gut microbiota is related to the occurrence and development of various diseases (6, 7). Recently, large-scale observational epidemiological studies and animal studies have suggested that microbial disorder and hypertension might be causally related (8, 9). There may be some connection between *Firmicutes/Bacteroidetes* ratio and blood pressure (10). Studies suggest that changes in the gut microbiota have been involved in the pathogenesis of diabetes (11). Increasing evidence suggests that the microbial ecosystem is associated with the progression of multiple cancers, such as prostate cancer, pancreatic cancer, and liver cancer (12). Gut microbiota imbalance can affect the production of short-chain fatty acids (SCFAs), bile acid metabolism, and intestinal mucosa, leading to the occurrence of diseases (13). Therefore, it is important to maintain the intestinal microenvironment for human health.

Whole grain is characterized by the retention of the endosperm, germ, and bran found in whole grain (WG). WG is a good source of dietary fiber, protein, B vitamins, vitamin E, minerals (such as selenium, copper, and magnesium), flavonoids, and polyphenols (14–16). Numerous studies have shown that they can help to reduce the risk of many diseases, such as overweight (OW)/obesity, cardiovascular disease, type 2 diabetes, intestinal disease, and certain cancers, as compared to refined grains. Several studies have shown that WG can significantly alter the diversity, structure, and function of the gut microbiota. A recent study shows that WG can increase the abundances of Ruminococcaceae_UCG-014, Ruminococcaceae_UCG-014, Ruminocostridium_9, and Ruminococcaceae_NK4A214_group (17). This study explored the possible effects of WG on the health of the elderly individuals from the perspective of gut microbiota.

## Materials and Methods

### Data Sources

The American Gut Program (AGP) collected 25,376 samples through voluntary and self-reported methods that included basic information (age, gender, height, and weight) and lifestyle-related information. The collection, sequencing, and quality control of samples were carried out according to the standards of the Earth Microbiome Project.

American Gut Program sequencing data had been stored in the Sequence Read Archive (SRA) database in SRA file format (https://www.ncbi.nlm.nih.gov/sra/), and the registration number was PRJEB11419. AGP questionnaire results could also be downloaded from the SRA database.

In the study, samples were strictly screened and matched. Those who did not fill in the basic information in the questionnaire, who showed serious diseases, who received antibiotic treatment within 6 months, and who had been traveling recently were excluded. Non-fecal samples were also excluded. In addition, according to the sequencing quality, samples with a sequencing depth of <8,000 were excluded. Finally, we found 70 elderly individuals who daily fed on/ate WG (DWG) and matched 70 elderly individuals who never fed on/ate WG (NWG) according to gender, body mass index (BMI), and age (Table 1 and Supplementary Table S1).

**Table 1:** Demographic and anthropometric characteristics in the elderly individuals of the daily whole-grain (WG) diet and the never whole grain diet.

	**DWG**	**NWG**	**Chi-square**	***p*** **-Value**
Elderly	66.74 ± 0.7918	67.01 ± 0.7918		
Total number	70	70		
BMI	24.09 ± 0.7694	24.65 ± 0.7694		
Normal weight (number)	44	44		
Overweight (number)	26	26		
Gender
Female (number)	33	33		
Male (number)	37	37		
Country_residence
African American (number)	0	1	0.9930	0.3190
Asian or Pacific Islander (Number)	5	3	0.4731	0.4916
Caucasian (number)	60	64	0.06844	0.7936
Other (number)	5	1	2.559	0.1097
Hispanic (number)	0	1	0.9930	0.3190
Diet_type				
Omnivore	49	61	0.7348	0.3913
Vegan & Vegetarian	11	1	7.709	0.0055
Omnivores but do not eat red meat	5	4	0.1044	0.7466
Vegetarian but eat seafood	4	3	0.1361	0.7122
Not provided	1	1	0.000	>0.9999

### Converting SRA to FASTQ Format

The files in the SRA database were in SRA format and needed to be converted to FASTQ format for further analysis. The SRA Toolkit tool was employed for format conversion.

### Data Processing

The FASTQ format file was used for flora analysis. This process used the QIIME2 software. The FASTQ format file was first packaged into a demux.qza file. Then the Deblur plug-in was used to perform denoising and Operational Taxonomic Unit (OTU) clustering and generate result files with representative sequences, feature tables, and sample statistics. Unrooted trees are obtained through the “qiime-phylogeny-align-to-tree-mafft-fastree” plug-in. Alpha diversity is obtained through the “qiime diversity alpha sparse” plugin and converted to qzv files. QZV file can view OTU number and Shannon index through the browser.

Then, we compared it with the Greengenes database (version 13.8), and the comparison method was UCLUST, identity 0.9, to complete the classification of bacteria. In addition, we deleted the bacteria with low distribution (which only existed in <1% of the samples) for subsequent analysis.

Phylogenetic Investigation of Communities by Reconstruction of Unobserved States (PICRUSt2) was used to predict functional abundance based on marker gene sequences.

### Statistical Analysis

Statistical Analysis of Metagenomic Profiles (STAMP 2.1.3) was used to analyze microbial data, such as microbial community composition and function. Adjusted *p*-values were calculated by Benjamin Hochberg's false discovery rate (FDR) method and used to analyze significant differences (*p*_adj_ < 0.05).

## Result

The present study detected the effects of WG on the gut microbiota of the elderly individuals. As shown in Table 1, there is no significant difference in age, BMI, and gender between the DWG and NWG dietary groups.

### Effects of WG on Gut Microbiota in the Elderly Individuals

**Figure 1:**
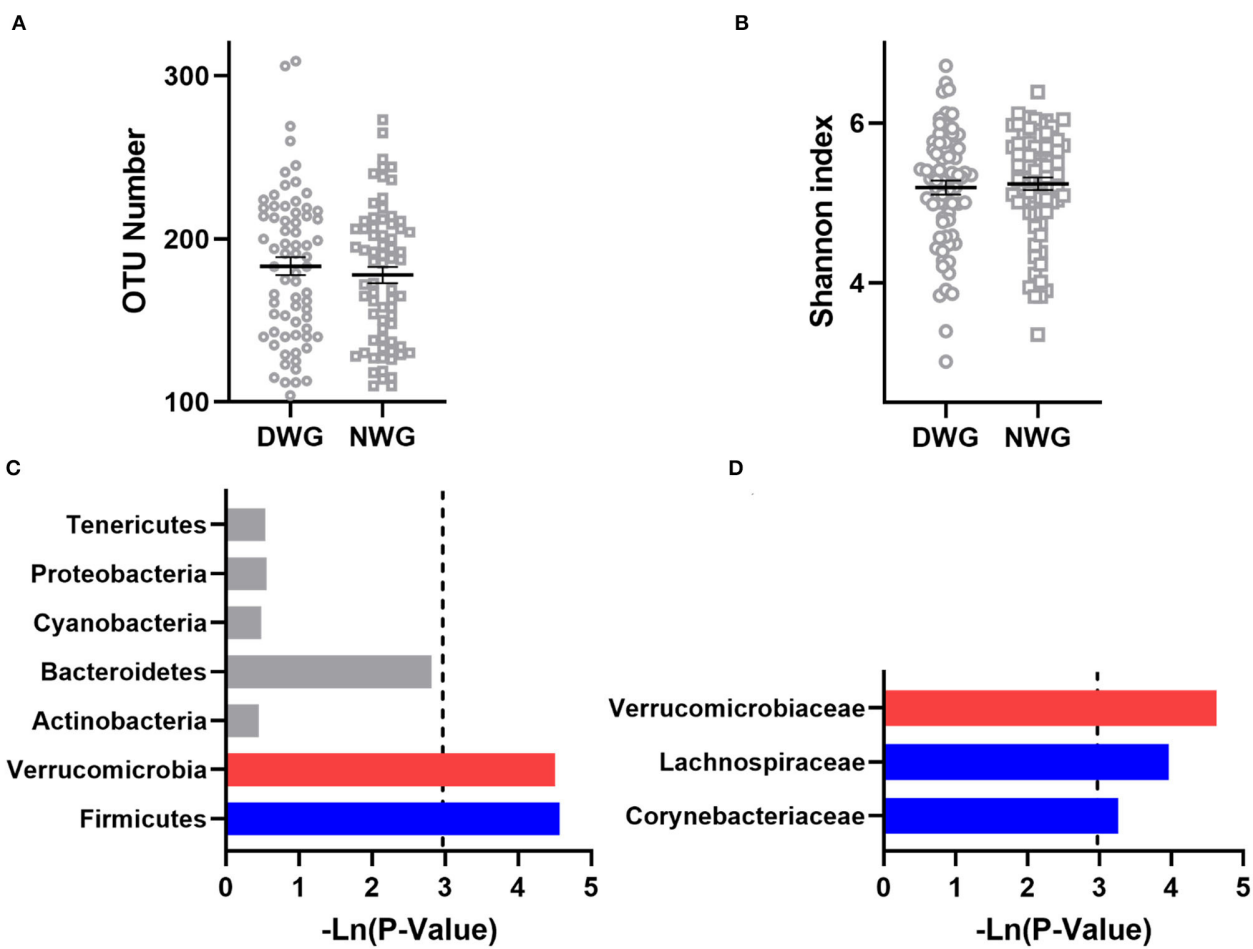
The effect of whole-grain (WG) diet on the gut microbiota composition of elderly individuals. **(A)** Number of OTUs, **(B)** Shannon index, **(C)** phylum level, and **(D)** family level. The red bar represents a significant increase, while the blue bar represents a significant decrease.

To detect the effect of WG on the gut microbiota of the elderly individuals, α-diversity was analyzed (Figures 1A,B). The OTU numbers of DWG and NWG were 183.2 and 177.9, respectively (*p* > 0.05), and the Shannon index values were 5.192 and 5.239, respectively (*p* > 0.05). The results showed that the microbial diversity in the elderly individuals was not affected by WG.

The effect of WG on the gut microbiota composition was further analyzed (Figures 1C,D). At the phylum level, when compared with NWG, the relative abundance of *Verrucomicrobia* was increased in the DWG, while the relative abundance of bacteria *Firmicutes* was decreased. At the family level, the relative abundance of *Verrucomicrobiaceae* was increased, while that of *Lachnospiraceae* and *Actinomycetaceae* was decreased.

**Figure 2:**
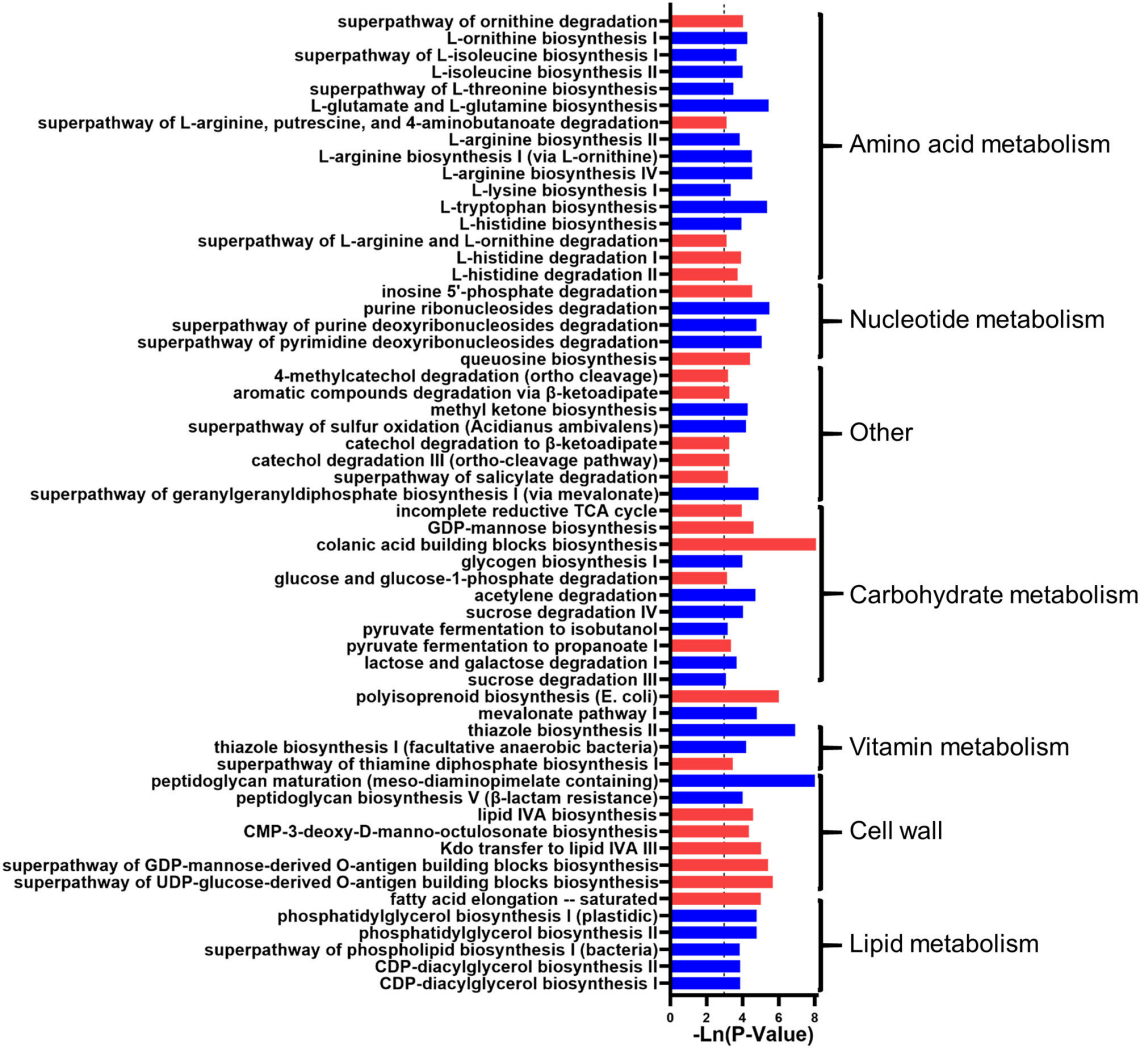
The effect of whole-grain (WG) diet on the gut microbiota function of elderly individuals. The significant effect of the WG diet on microbial metabolism pathways. The red bar represents a significant increase, while the blue bar represents a significant decrease.

In addition, the microbial function predicted that 58 metabolic pathways had significant changes, i.e., 16 amino acid metabolism-related pathways, five nucleotide metabolism-related pathways, one biological oxidation pathway, 11 carbohydrate metabolism-related pathways, two terpenoid synthesis pathways, three vitamins related pathways, seven cell wall-related metabolic pathways, and six lipid metabolism pathways. Generally, the metabolism of amino acids, nucleotides, biological oxidation, sugar, vitamins, and lipids was inhibited in the DWG group (Figure 2).

### Effects of WG on the Gut Microbiota of Normal Weight (NW) Group and OW Group in the Elderly Individuals

**Figure 3:**
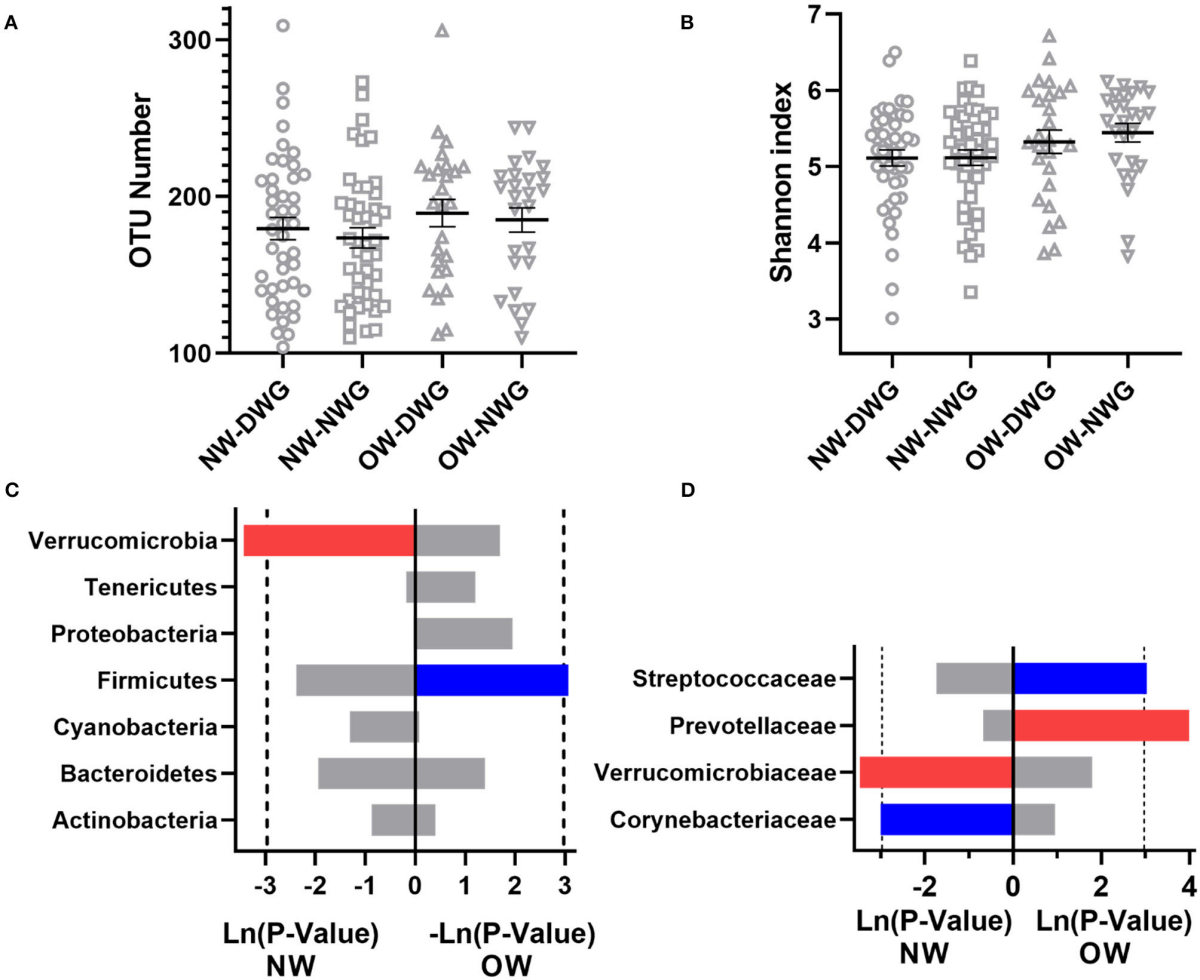
The effect of whole-grain (WG) diet on the gut microbiota composition of female and male elderly individuals. **(A)** Number of OTUs, **(B)** Shannon index, **(C)** phylum level, and **(D)** family level. The red bar represents a significant increase, while the blue bar represents a significant decrease.

To further explore the effect of WG on the gut microbiota of different BMI elderly individuals, the samples were divided into NW and OW (BMI > 25) groups. As shown in Figure 3, the OTU values of NW_DWG, NW_NWG, OW_DWG, and OW_NWG are 179.5, 173.7, 189.5, and 185.1, respectively (*p* > 0.05), and the Shannon index values are 5.113, 5.117, 5.326, and 5.446, respectively (*p* > 0.05). In addition, when compared with the NW group, both OTU and Shannon index of the OW group showed an upward trend, which indicated that the intestinal microbial diversity of the elderly individuals changed with the increase in BMI.

Next, the relative abundance of microbial components was detected at the phylum and family level (Figures 3C,D). At the phylum level, the relative abundance of *Verrucomicrobia* in the NW_DWG group was significantly higher than that in the NW_NWG group. Compared with the OW_NWG group, the relative abundance of *Firmicutes* was significantly lower in the OW_DWG group. At the family level, the relative abundance of *Verrucomicrobiaceae* was increased, while the relative abundance of Actinomycetaceae was decreased in the NW_DWG group, when compared with the NW_NWG group. The relative abundance of *Streptococcaceae* was decreased significantly, while the relative abundance of *Prevotellaceae* was increased significantly in OW_DWG, when compared with OW_NWG.

**Figure 4:**
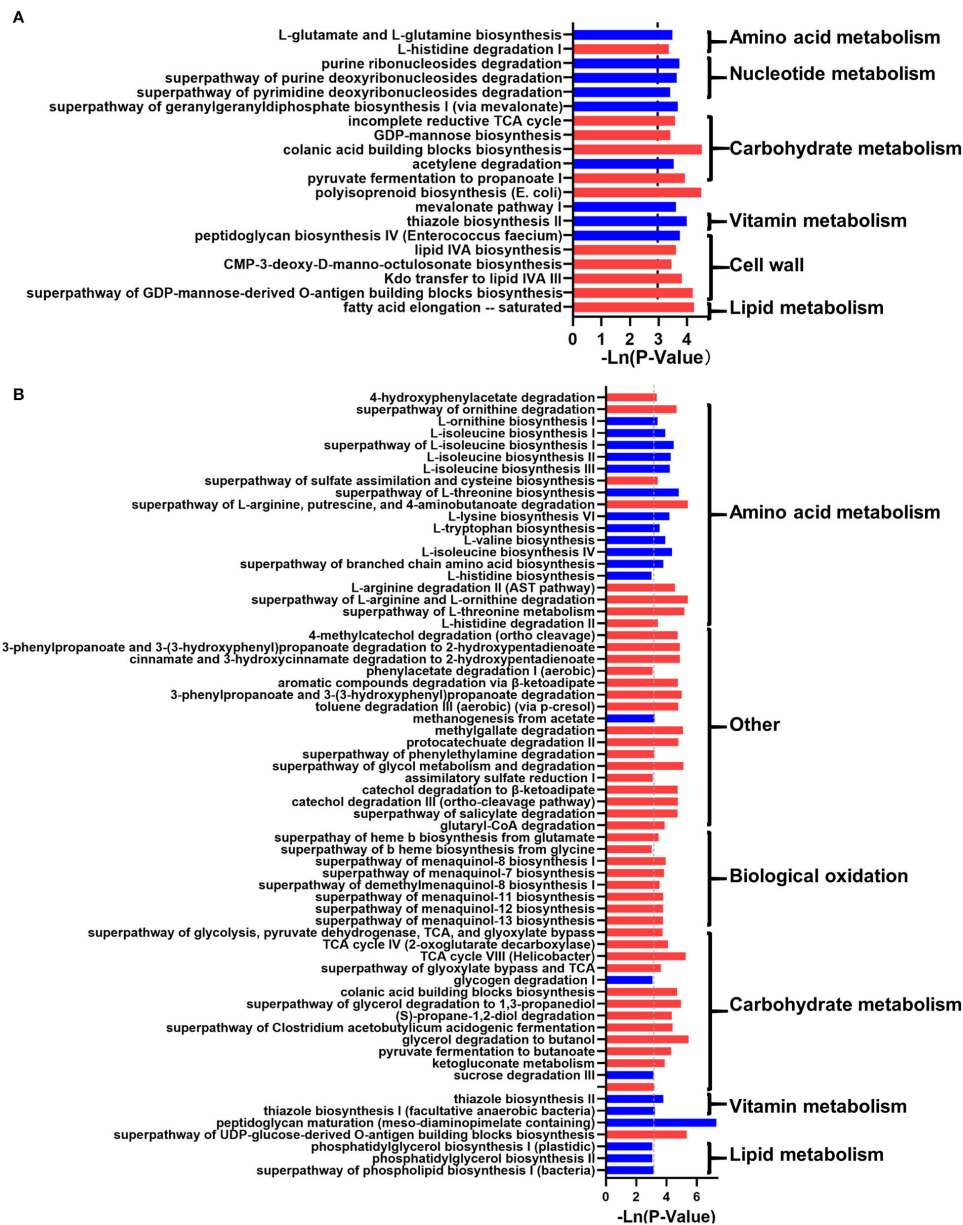
The effect of the whole-grain (WG) diet on the gut microbiota function of female and male individuals. The significant effect of the WG diet on microbial metabolism pathways in **(A)** female and **(B)** male individuals. The red bar represents a significant increase, while the blue bar represents a significant decrease.

In addition, the prediction of microbial function showed that 20 and 66 metabolic pathways were changed significantly (*p* < 0.05; Figure 4). In NW_DWG, the metabolic pathways related to sugars, cell walls, and lipids were significantly increased, while the metabolism of nucleotides, biological oxidation, and vitamins was significantly decreased. In OW_DWG, the metabolic pathways of amino acids, vitamins, and lipids were significantly decreased, while those of SCFAs, biological oxidation, sugars, and other substances were significantly increased.

### Effects of WG on the Gut Microbiota of Female and Male Elderly Individuals

**Figure 5:**
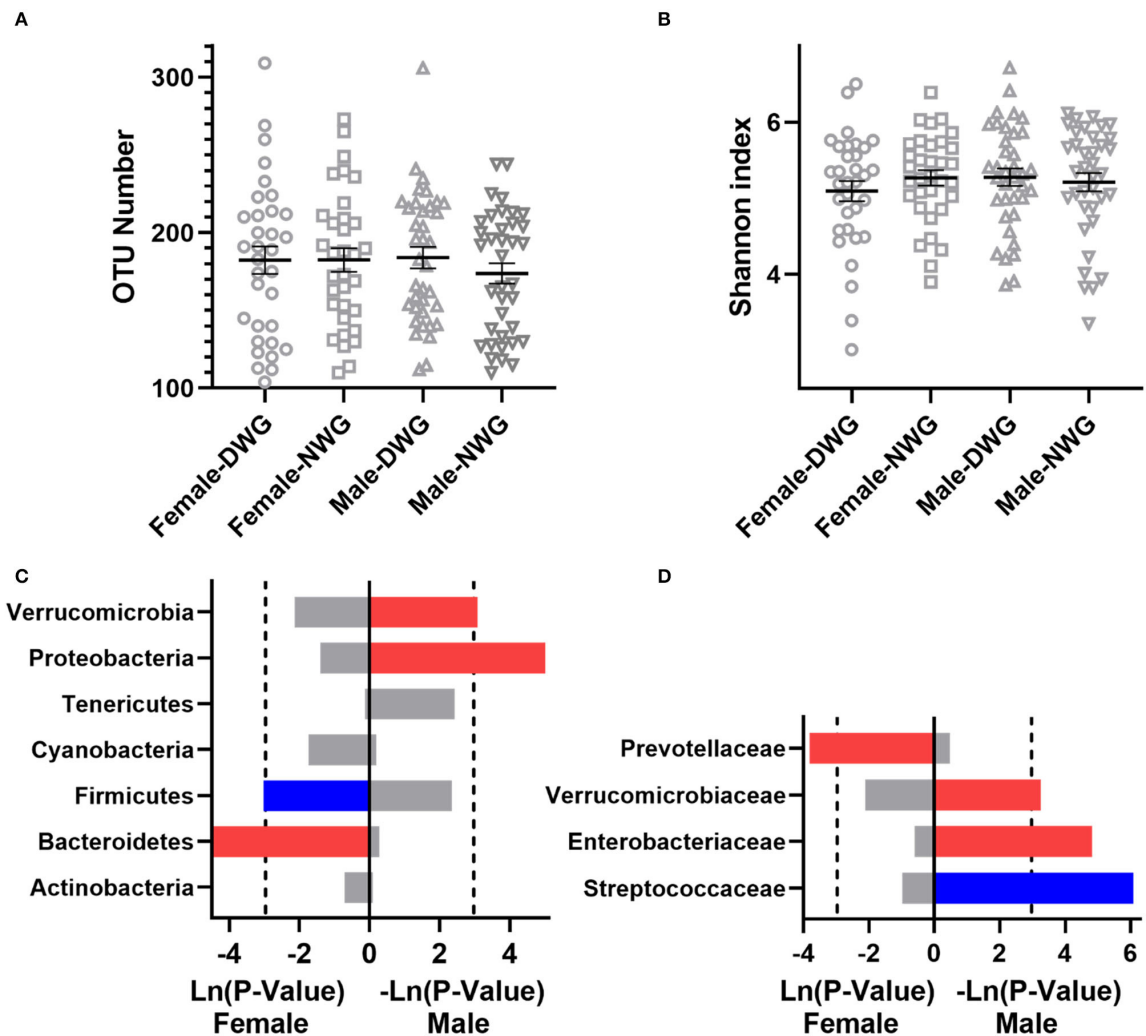
The effect of whole-grain (WG) diet on the gut microbiota composition of normal-weight and overweight elderly individuals. **(A)** Number of OTUs, **(B)** Shannon index, **(C)** phylum level, and **(D)** family level. The red bar represents a significant increase, while the blue bar represents a significant decrease.

Furthermore, the effects of WG on the gut microbiota of female and male elderly individuals were analyzed. The results showed that the DWG did not affect microbial α-diversity of male and female elderly individuals (Figures 5A,B). At the phylum level, the relative abundance of *Firmicutes* was significantly reduced, while that of *Bacteroidetes* was significantly increased in female DWG, when compared with that of female NWG. The relative abundance of *Verrucomicrobia* and *Proteobacteria* was significantly increased in Male_DWG, when compared with Male_NWG. At the family level, the relative abundance of *Prevotellaceae* was significantly increased in female DWG, when compared with that of the female NWG group. The relative abundance of *Streptococcaceae* was significantly decreased, while that of *Verrucomicrobiaceae* and *Enterobacteriaceae* was significantly increased in Male_DWG, when compared with that of Male_NWG (Figures 5C,D).

**Figure 6:**
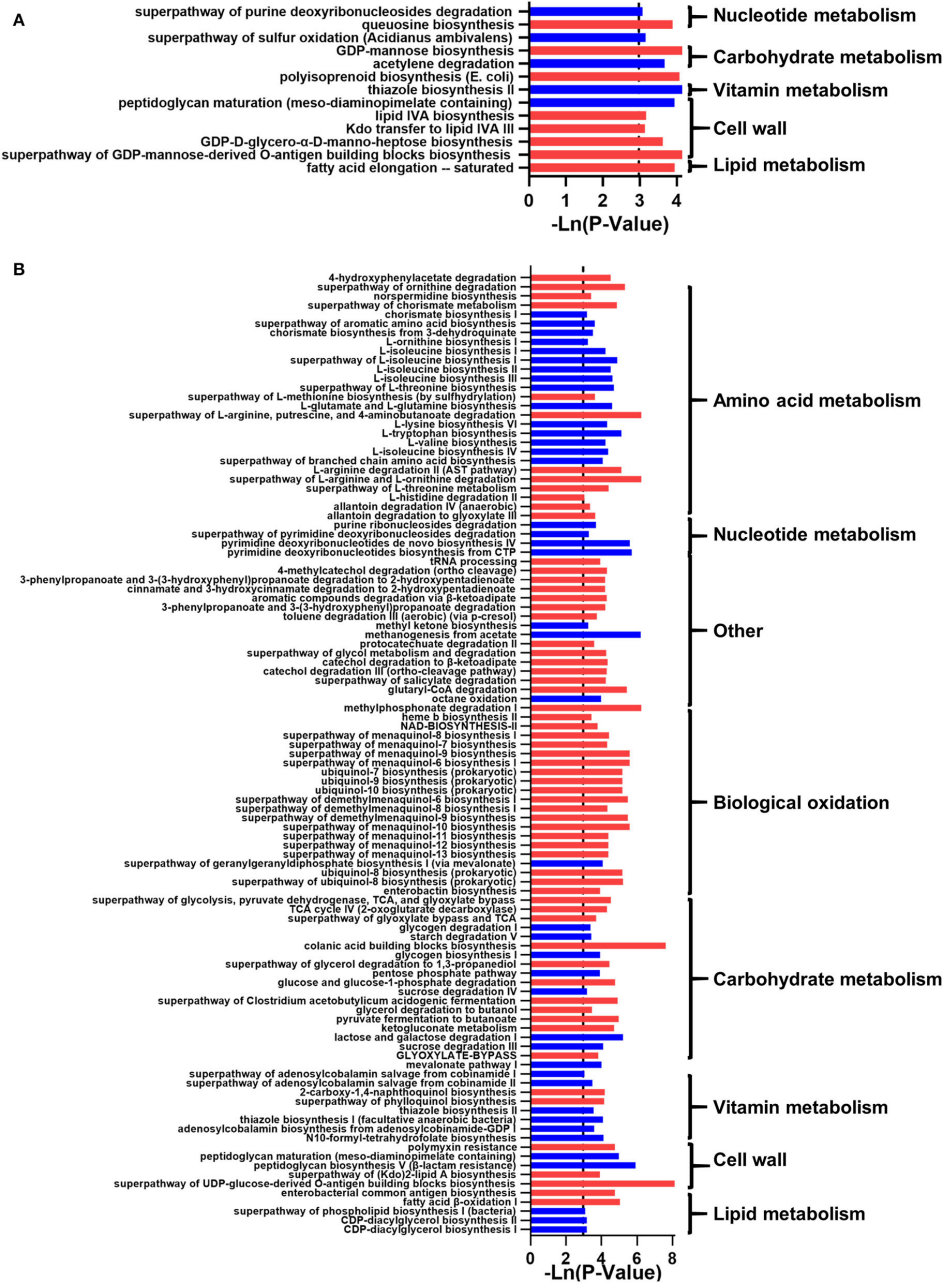
The effect of the whole-grain (WG) diet on the gut microbiota function of normal weight and overweight individuals. The significant effect of the WG diet on microbial metabolism pathways, **(A)** normal-weight and **(B)** overweight individuals. The red bar represents a significant increase, while the blue bar represents a significant decrease.

In addition, functional predictions indicated that 13 and 105 related metabolic pathways were significantly changed in Female_DWG and Male_DWG (*p* < 0.05; Figure 6). In Female_DWG, the related metabolic pathways of terpenoids, cell walls, and lipids were significantly increased, and the metabolism of vitamins is significantly reduced. In Male_DWG, the metabolic pathways related to SCFA, nucleic acid bio-oxidation, and lipo-sugar were significantly increased, while the related metabolic pathways of amino acids, nucleotides, terpenoids, vitamins, and lipids were significantly reduced.

## Discussion

Studies have shown that diet can directly affect the diversity and composition of human gut microbiota (18, 19). WG has a significant effect on the gut microbiota of obesity, DM, and hypertension (20–22). The present study used the data from the AGP to analyze the effect of WG on the gut microbiota of the elderly individuals. To simplify the analysis model, we screened out the microbial data of the elderly individuals who never and/or daily feed on WG for analysis. The results showed that the WG did not affect the microbial α-diversity in the elderly individuals, but had a significant effect on the microbial composition and function.

Studies have shown that WG can significantly affect microbial community composition (23, 24). Wheat WG could significantly increase *Bifidobacteria, Lactobacilli*, and *Prevotella* and significantly decrease *Dialister, Bifidobacterium, Blautia*, and *Collinsella* (25, 26). Quinoa could alleviate *dysbiosis* remarkably by increased species richness and diversity, decreased phylum *Proteobacteria*, and genera Escherichia and *Peptoclostridium* (23). A 6-week randomized controlled trial shows that WG can effectively reduce the weight of OW, and it is related to *Prevotella* abundance (27). This may be related to the WG-rich diet reducing systematic low-grade inflammation and keeping the gut microbiota stable (28). WG significantly decreased *Firmicutes/Bacteroidetes* ratio, *Blautia, Roseburia, Bifidobacterium*, and *Dialister* (29, 30). The present study showed that DWG could decrease the abundance of *Firmicutes, Lachnospiraceae*, and *Actinomycetaceae* and increase the abundance of *Verrucomicrobia* and *Verrucomicrobiaceae*. Therefore, the present study and previous research have shown that WG intake can reduce the *Firmicutes/Bacteroidetes* ratio, and this study provides more gut bacteria associated with WG intake. Meanwhile, the study provides more intestinal microbes related to WG intake.

Studies have shown that the components of WG (dietary fiber, phenols, and B vitamins) can affect the gut microbiota. High dietary fiber can effectively improve the diversity and composition of gut microbiota, such as *Clostridiales*, which participate in carbohydrate utilization *via* polysaccharide degradation (31). The type and composition of dietary fiber from WG can affect the production of SCFA, such as arabinoxylan mixed with β-glucan, which can affect butyrate production. WG is high in phenolic acids, mainly covalently bound to fiber (32). The gut microbiota could release covalently bound phenolics from fiber and undergo metabolic conversion. Biotransformation of phenolic compounds may favor specific bacterial species, which in turn affect dietary fiber degradation pathways (32). In addition, B vitamins also play an important role in the regulation of gut microbiota (33). The present study found that WG not only affected the composition of gut microbiota but also predicted that WG could achieve beneficial effects by modulating the effect of gut microbiota on the metabolism of amino acids, carbohydrates, lipids, and vitamins.

Being OW is an important factor threatening the health of the elderly people (34). Studies have shown that the microbial composition of OW individuals is significantly affected (33, 35). Increased *Streptococcaceae* is generally considered to be associated with diseases, such as diabetes, obesity, and hypertension (36–38). Moreover, gut microbiota rich in *Prevotella* can improve glucose metabolism, potentiate weight loss, and reduce cholesterol levels (39–41). Researchers believe that the high diversity of *Prevotella* can enhance the fermentation ability of the microbiota, which is conducive to human health (42). *Akkermansia* in *Verrucomicrobiaceae* is inversely associated with obesity, diabetes, and cardiovascular disease (43). Consistent with previous studies, the study found that being OW was an important factor affecting the gut microbiota of the elderly people. The level of *Firmicutes* and *Streptococcaceae* was decreased, while the level of *Prevotella* was increased in the OW_DWG group. In addition, *Verrucomicrobiaceae* was significantly increased in the NW_DWG group. In general, WG diets can increase the abundance of probiotics and inhibit the growth of disease-related microbes for both NW and OW individuals.

Several studies have shown that there are significant differences in the gut microbiota of men and women (44, 45). This may be due to the male and female receiving different hormonal disturbances or different dietary patterns (46, 47). Studies have shown that the gut microbiota of men and women respond differently to the same diet and pharmacological interventions (48–50). A study showed an increased abundance of *Lactobacillus, Alistipes, Lachnospira*, and *Clostridium* in male mice but not in female mice when fed with HFD (48). A recent study showed that a diet of fructooligosaccharides had differential effects on SCFAs in the gut of male and female rats, while an increased abundance of *Bacteroides* was found only in female rats (49). A tuna oil and algae oil mixture treatment resulted in different gut microbial composition alterations in different sexes (51). The present study showed that WG significantly increased Verrucomicrobia and decreased *Streptococcaceae* in men, while significantly suppressing the *Firmicutes* to *Bacteroidetes* ratio and increasing *Prevotellaceae* in women. This study highlights the inconsistencies in the effect of WG on the gut microbiota in the male and female elderly individuals.

However, this study has some shortcomings due to AGP's project design. For example, all data are self-reporting, and its authenticity cannot be traced back. Although AGP collected more than 25,000 samples, there is little data available. Specific to the present study, we only matched 70 samples. In addition, the sample data come from the Caucasian population, and there is a lack of data on different races. These were worthy of further exploration in future research.

## Conclusion

This study analyzed the effect of WG on the gut microbiota of the elderly people. It was found that although WG did not change the α-diversity of the microbes, it affected the abundance of *Verrucomicrobia* and *Firmicutes*. In addition, for the WG, different weights and gender have different outcomes. WG affected the abundance of *Verrucomicrobia, Verrucomicrobiaceae*, and *Corynebacteriaceae* bacteria in NW people and affected the abundance of *Firmicutes, Streptococcaceae*, and *Prevotellaceae* bacteria in OW people. WG affected the abundance of *Firmicutes, Bacteroidetes*, and *Prevotellaceae* in men and *Verrucomicrobia, Proteobacteria, Verrucomicrobiaceae*, and *Streptococcaceae* bacteria in women. In general, WG can increase the abundance of beneficial bacteria and partially inhibit the growth of harmful bacteria.

## Data Availability Statement

The original contributions presented in the study are included in the article/Supplementary Material, further inquiries can be directed to the corresponding authors.

## Author Contributions

GD contributed to funding acquisition, investigation, methodology, project administration, resources, and writing—original draft and editing. YZ and ZC contributed to the software. JL contributed to the conceptualization of formal analysis. PF contributed to writing—original drafts and editing. All authors contributed to the article and approved the submitted version.

## Funding

This work was funded by the National Natural Science Foundation of China (No. 81960672 to GD), Natural Science Foundation of Hainan Province (821MS0830 to PF), and had no role in the study design, data collection analysis, decision to publish, or preparation of the manuscript.

## Conflict of Interest

The authors declare that the research was conducted in the absence of any commercial or financial relationships that could be construed as a potential conflict of interest.

## Publisher's Note

All claims expressed in this article are solely those of the authors and do not necessarily represent those of their affiliated organizations, or those of the publisher, the editors and the reviewers. Any product that may be evaluated in this article, or claim that may be made by its manufacturer, is not guaranteed or endorsed by the publisher.
